# The effects of cancer therapies on physical fitness before oesophagogastric cancer surgery: a prospective, blinded, multi-centre, observational, cohort study

**DOI:** 10.3310/nihropenres.13217.1

**Published:** 2021-06-16

**Authors:** Malcolm A. West, Zachos Anastasiou, Gareth Ambler, Lisa Loughney, Michael G. Mythen, Thomas Owen, Gerard Danjoux, Denny Z.H. Levett, Peter M.A. Calverley, Jamie J. Kelly, Sandy Jack, Michael P.W. Grocott, Fit4Surgery Consortium

**Affiliations:** 1 Academic Unit of Cancer Sciences, Faculty of Medicine, University of Southampton, Southampton, SO16 6YD, UK; 2 Integrative Physiology and Critical Illness Group, Clinical and Experimental Sciences, Faculty of Medicine, University of Southampton, Southampton, SO16 6YD, UK; 3 Acute Perioperative and Critical Care Research Group, Southampton NIHR Biomedical Research Centre, University Hospital Southampton NHS Foundation Trust, Southampton, SO16 6YD, UK; 4 Department of Statistical Science, University College London, London, W1T 7PJ, UK; 5 Centre for Anaesthesia, Institute of Sport Exercise and Health, University College London Hospitals NIHR Biomedical Research Centre, London, W1T 7HA, UK; 6 Department of Critical Care and Anaesthesia, Lancashire Teaching Hospitals NHS Foundation Trust, Lancashire, PR7 1PP, UK; 7 Department of Critical Care and Anaesthesia, The James Cook University Hospital, Middlesborough, TS4 3BW, UK; 8 Department of Respiratory Research, University of Liverpool, University Hospitals Aintree, Liverpool, L9 7AL, UK; 9 Department of Upper Gastro-intestinal Surgery, University Hospital Southampton NHS Foundation Trust, Southampton, SO16 6YD, UK

**Keywords:** O2 diffusion during exercise, Pre-operative evaluation: American College of Cardiology Guidelines

## Abstract

**Background:**

Neoadjuvant cancer treatment is associated with improved survival following major oesophagogastric cancer surgery. The impact of neoadjuvant chemo/chemoradiotherapy on physical fitness and operative outcomes is however unclear. This study aims to investigate the impact of neoadjuvant chemo/chemoradiotherapy on fitness and post-operative mortality.

**Methods:**

Patients with oesophagogastric cancer scheduled for chemo/chemoradiotherapy and surgery were recruited to a prospective, blinded, multi-centre, observational cohort study. Primary outcomes were changes in fitness with chemo/chemoradiotherapy, measured using cardiopulmonary exercise testing and its association with mortality one-year after surgery. Patients were followed up for re-admission at 30-days, in-hospital morbidity and quality of life (exploratory outcomes).

**Results:**

In total, 384 patients were screened, 217 met the inclusion criteria, 160 consented and 159 were included (72% male, mean age 65 years). A total of 132 patients (83%) underwent chemo/chemoradiotherapy, 109 (71%) underwent chemo/chemoradiotherapy and two exercise tests, 100 (63%) completed surgery and follow-up. A significant decline in oxygen uptake at anaerobic threshold and oxygen uptake peak was observed following chemo/chemoradiotherapy: -1.25ml.kg ^-1^.min ^-1^ (-1.80 to -0.69) and -3.02ml.kg ^-1^.min ^-1^ (-3.85 to -2.20); p<0.0001). Baseline chemo/chemoradiotherapy anaerobic threshold and peak were associated with one-year mortality (HR=0.72, 95%CI 0.59 to 0.88; p=0.001 and HR=0.85, 0.76 to 0.95; p=0.005). The change in physical fitness was not associated with one-year mortality.

**Conclusions:**

Chemo/chemoradiotherapy prior to oesophagogastric cancer surgery reduced physical fitness. Lower baseline fitness was associated with reduced overall survival at one-year. Careful consideration of fitness prior to chemo/chemoradiotherapy and surgery is urgently needed.

## Plain language summary

Background: Cancer treatments such as chemotherapy and radiotherapy given to people with oesophageal and gastric cancer (also known as cancer of the food pipe/stomach) before surgery can improve survival. However, the impact such treatments have on fitness and recovery after surgery is unclear. The aim of this research was to understand the impact cancer treatments has on fitness and any complications after surgery.

Methods: Patients with oesophageal and gastric cancer (also known as cancer of the food pipe/stomach) who were being treated by cancer treatment and surgery were recruited from different hospitals in the UK. All participants were asked to undertake an exercise test to measure fitness and fill out questionnaires to measure quality of life before and after cancer treatment. Complications patients experienced after surgery, the number of patients who had to be readmitted to hospital 30 days after surgery and one-year survival was recorded.

Results: A total of 160 consented to participate in this study and 159 were included in the study (72% male, average age 65 years). In total, 132 patients (83%) had cancer treatment, 109 (71%) had cancer treatment and the two exercise tests and 100 (63%) had surgery and were followed-up after surgery. Study findings show that fitness reduced after cancer treatment. Patient’s fitness levels at the start of the study (or before cancer treatment) were linked to one-year survival. The fall in fitness after cancer treatment was not linked to death at the one-year follow-up.

Conclusion: Cancer treatments before oesophageal and gastric cancer reduce fitness. Patients with a lower fitness level before cancer treatment had a reduced overall survival at one-year. Careful consideration of fitness prior to such cancer treatments and surgery is urgently needed.

## Introduction

For patients with locally advanced oesophagogastric (OG) cancer, multimodal therapy incorporating surgery and neoadjuvant chemotherapy or combined chemoradiotherapy (referred to as chemo/chemoradiotherapy herein) offers improved survival over surgical therapy alone ^1– 4^. However, improvement in overall survival may come at the cost of increased treatment toxicity and mortality in some patients ^5, 6^.

Physical fitness assessed objectively using Cardiopulmonary Exercise Testing (CPET) may be the best functional predictor of complications following major surgery ^7^ and is increasingly being adopted in the perioperative setting to guide perioperative care and decision-making ^8, 9^. Preliminary data from our group and others suggest that chemo/chemoradiotherapy before OG cancer surgery result in a clinically important reduction in physical fitness (oxygen update (VO _2_) at anaerobic threshold (AT) and VO _2 _peak) ^10– 13^. In a small, single-centre, pilot, unblinded study, we previously reported that low baseline physical fitness (VO _2_ at AT and VO _2 _peak) was associated with reduced one-year survival in patients completing chemotherapy and surgery, but not in patients who did not complete chemotherapy ^10^.

The aims of this study were to investigate the impact of chemo/chemoradiotherapy on fitness (VO _2_ at AT and VO _2 _peak), mortality (at one-year after surgery) and post-operative outcomes (Post-Operative Morbidity Survey and EQ-5D-5L). In this prospective, multi-centre, blinded study, we set out to validate the hypothesis that chemo/chemoradiotherapy was associated with reduced physical fitness and that this change in physical fitness (relative change and change in risk stratification category) would be associated with all-cause mortality at one-year. Further, we explored the hypotheses that reduced fitness following chemo/chemoradiotherapy was associated with increased post-operative morbidity and worse patient reported outcomes.

## Methods

### Study design

This prospective study involved participants with OG cancers scheduled for chemo/chemoradiotherapy followed by elective resection with curative intent. The study protocol, methods and statistical analysis plan are available in open access format ^14^. This study was funded by the National Institute for Health (NIHR), Research for Patient Benefit Programme (PB-PG-0609-18262). The research protocol was registered with clinicaltrials.gov (NCT01325883 - 30th March 2011) and approved by the Dyfed Powys Research Ethics Committee (11/WA/0072). Written informed consent was obtained from all subjects. The study is described according to the STROBE statement ^15^.

### Recruiting hospitals

The study was conducted in four NHS hospitals in England: University Hospital Southampton (Southampton), University Hospital Aintree (Aintree), Lancashire Teaching Hospital (Preston) and South Tees Hospital (South Tees). A study management board and independent data and safety monitoring committee oversaw the project. 

### Eligibility criteria

Briefly, patients with a histologically confirmed, potentially curable (able to undergo chemo/chemoradiotherapy followed by curative elective resection) adenocarcinoma, squamous, or mucinous/ undifferentiated carcinoma of the oesophagus, oesophagogastric junction (i.e. tumours involving both the cardia and the oesophagus on endoscopy) or stomach were eligible for inclusion. Eligible patients were ≥18 years of age, had a World Health Organization (WHO) performance status score of ≤2, and had adequate hematologic, renal, hepatic and pulmonary function, as well as no history of other cancer or previous chemo/chemoradiotherapy. The study excluded patients who were unable to give informed consent, had non-resectable disease, were unable to perform CPET due to known contra-indication (e.g. lower limb dysfunction), or who declined planned surgery or neoadjuvant cancer treatments. Eligible patients were staged according to a pre-determined protocol. All patients underwent pre-treatment staging based on a pre-determined protocol. This included a medical history, physical examination, pulmonary function tests, routine hematologic and biochemical test, esophago-gastric endoscopy with histologic biopsy +/- endoscopic ultrasound, computer tomography of the neck, chest and abdomen, 18F-fluorodeoxyglucose positron-emission tomography and in special circumstances external ultrasonography of the neck, with fine-needle aspiration of lymph nodes when cancer was suspected. Re-staging was undertaken using computer tomography of the chest and abdomen and 18F-fluorodeoxyglucose positron-emission tomography +/- laparoscopy in selected cases. Radiological responses post-chemo/chemoradiotherapy were based on definitions outlined by the RECIST version 1.1 criteria for solid target lesions ^16^.

### Study recruitment

All potentially eligible patients were identified at multi-disciplinary meetings and approached with written information. All patients provided written informed consent.

### Outcome measures

***Fitness.*** CPET was used to assess physical fitness before and following completion of chemo/chemoradiotherapy (approximately four weeks following completion of chemo/chemoradiotherapy, immediately before planned surgery) and CPET was conducted according to a published protocol ^17, 18^. All CPETs were performed using identical software and hardware at each recruitment site using an electromagnetically braked cycle ergometer (Ergoline 2000), a 12-lead ECG, non-invasive blood pressure measurement and pulse oximetry, and a metabolic cart (Geratherm Respiratory GmbH, Love Medical Ltd). CPET allowed for the derivation of anaerobic threshold (AT) using the modified V-Slope method. The modified V-Slope method identifies the anaerobic threshold as the tangential breakpoint in the rate of change of VCO _2 _relative to VO _2 _(oxygen uptake – carbon dioxide output) from the line of unity (‘line of one’) during the incremental stage of the exercise test. CPET was independently reported by two independent experienced observers (SJ, DL) blinded to CPET timepoint and clinical outcomes, with a third adjudicator (MAW) if >5% variance in VO _2 _at AT was observed. All cancer multi-disciplinary team members including the treating surgeon, anaesthetist, oncologist and peri-operative teams were blind to all CPET data.

***Health-related quality of life (HRQol)***. HRQoL was measured using patient reported outcome measure (EQ-5D-5L ^19^) questionnaire before and following completion of chemo/chemoradiotherapy (approximately four weeks following completion of chemo/chemoradiotherapy, immediately before planned surgery).

***Post-operative outcome.*** All patients were followed-up (by staff blinded to CPET results), using the Post-Operative Morbidity Survey (POMS) ^20^ at day 3, 5, 8 and 15 post-operatively. Length of hospital stay and critical care length of stay was calculated by subtracting the discharge date from the admission date. The Revised Cardiac Risk Index (RCRI) ^21^ was calculated preoperatively and the O-POSSUM score was completed postoperatively ^22^.

### Neoadjuvant chemo/chemoradiotherapy

We did not attempt to standardise chemo/chemoradiotherapy regimes. Chemotherapy regimens included: Epirubicin, Oxaliplatin, Capecitabine (EOX); Epirubicin, Cisplatin, Capecitabine (ECX) ^23^; Epirubicin, Cisplatin, 5-Fluorouracil (ECF) ^24^, chemotherapy as part of the STO3 trial – ECX or ECX + Bevacizumab ^25^, chemotherapy as part of the OEO5 trial – ECX or Cisplatin and 5-Fluorouracil ^26^; chemoradiotherapy as part of the CROSS trial – Carboplatin, Paclitaxel with concurrent radiotherapy ^27^; chemoradiotherapy as part of the NEOSCOPE trial – Oxaliplatin and Capecitabine or Carboplatin and Paclitaxel with concurrent radiotherapy and induction Oxaliplatin and Capecitabine chemotherapy ^28^; Herceptin, Cisplatin and Capecitabine; Capecitabine alone; and Cisplatin alone. CROSS style radiotherapy was administered at a total radiation dose of 41.4Gy given in 23 fractions of 1.8Gy each, with 5 fractions administered per week, starting on the first date of the first chemotherapy cycle. NEOSCOPE style radiotherapy was administered at a total radiation dose of 45Gy given in 25 fractions of 1.8Gy each, with 5 fractions administered per week, starting on the first date of the first chemotherapy cycle. All patients were treated with external beam radiation. Patients were closely monitored for toxic effects of chemo/chemoradiotherapy using the National Cancer Institute’s Common Terminology Criteria for Adverse Events, version 3.0 ^29^.

### Surgery

All patients underwent surgery within 4-6 weeks of chemo/chemoradiotherapy. Open, hybrid or fully minimal access approaches were used depending on patient characteristics and surgeon preference. A thoracoscopic assisted three-stage esophagectomy or an Ivor-Lewis esophagectomy based on tumour location was undertaken. A transthoracic approach was performed for tumours extending proximally to the tracheal bifurcation. For tumours involving the oesophagogastric junction, an Ivor-Lewis oesophagectomy resection was performed. Gastric tube reconstruction was the preferred technique for restoring intestinal continuity. Gastric surgery consisted of a radical resection of the primary tumour and at least a D1+ lymph node dissection.

### Follow-up

During the first year after surgery, patients were followed up for re-admission at 30-days post-operatively, all-cause mortality at 30-days and one-year post-operatively. Additional all-cause mortality follow-up was completed at five-year post-operatively. Patients completed an EQ-5D-5L questionnaire at 30-days and one-year post-operatively.

### Aims and objectives

Pre-defined primary aims briefly include:

1) observing changes in physical fitness (VO _2_ at AT and VO _2_ peak) following chemo/chemoradiotherapy, measured using CPET; and

2) interrogating the association of change in physical fitness following chemo/chemoradiotherapy and mortality one-year after surgery. This was evaluated in two ways: A) by evaluating the relative decrease in physical fitness associated with chemo/chemoradiotherapy and its association with mortality 1-year after surgery; and B) by evaluating whether a change in the risk stratification category (low risk VO _2_ at AT >14 ml.kg. ^-1^min ^-1^, medium risk VO _2_ at AT 11.0–14.0 ml.kg ^-1^.min ^-1^, high-risk VO _2_ at AT 8.0–10.9 ml.kg ^-1^.min ^-1^, highest risk VO _2_ at AT <8.0 ml.kg ^-1^.min ^-1^) following chemo/chemoradiotherapy would be associated with an increased one-year mortality following surgery when compared with those that do not change their risk stratification category. Similar interrogations were undertaken for mortality at 5-years after surgery. Risk categories were defined *a priori* ^14^.

The trial had several exploratory end points that are detailed in the study protocol ^14^. Briefly we explored:

1) the relationships between baseline/relative decrease in physical fitness (VO _2_ at AT and VO _2_ at Peak) following chemo/chemoradiotherapy and post-operative in-hospital morbidity (measured by the Post-Operative Morbidity Survey) and patient reported quality of life (measured by EQ-5D-5L);

2) the ability of less fit (VO _2_ at AT and VO _2_ at Peak) patients to tolerate chemo/chemoradiotherapy compared to patients with a higher fitness;

3) the relationship between patients who tolerate chemo/chemoradiotherapy poorly and an increase in post-operative outcomes (POMS, one-year mortality, EQ-5D); and

4) if patients undergoing chemoradiotherapy exhibit a greater decline in physical fitness (VO _2_ at AT and VO _2_ at Peak) compared to patients undergoing chemotherapy.

### Sample size calculation

Based on our previously published data ^10^ demonstrating a standard deviation of the differences in VO _2_ at AT values of 3.8 ml.kg ^.-1^.min ^-1^, we calculated that 152 patients were needed to detect a difference of 1.0 ml.kg ^.-1^.min ^-1^ of VO _2_ at AT using a paired t-test at the 5% significance level with 90% power (114 patients with 80% power), assuming the standard deviation of these differences is 3.8 ml.kg ^.-1^.min ^-1^. To detect a difference in one-year mortality rates of 23% (34% versus 11% - based on published pilot data ^10^) between the two VO _2_ at AT change groups [no change/deteriorate], we calculated that 104 patients were required using a chi-squared test at the 5% significance level with 80% power, assuming equal numbers of patients in both groups. 

### Statistical analysis

All data was inputted by double-data entry and validation was done according to procedures set out in the study data management and data validation plan overseen by the study management group.

Detailed statistical methods are described elsewhere ^14^. The primary analysis was a comparison of physical fitness (VO _2_ at AT) before and after chemo/chemoradiotherapy using a paired t-test. Distributional assumptions were assessed using a normal plot. Other fitness comparisons between independent patient groups were made using the two-sample t-test. The Cox proportional hazards model was used to investigate the relationship between ‘change in fitness’ and mortality within 5-year and the Kaplan-Meier plot was used to illustrate the survival of different patient groups. Multiple regression with backward elimination (at the 5% level) was used to investigate the relationship between post-chemo/chemoradiotherapy fitness and the various pre-chemo/chemoradiotherapy fitness variables. The predictive ability of both primary aim models to ascertain how prognostic the relative decrease in physical fitness was evaluated. Data was adjusted for baseline fitness and "penalized" regression models which contains more factors, even when the "Rule of 10" is not, met were used ^30^. Logistic regression was used to investigate the relationship between POMS morbidity and fitness. All analyses were performed with the statistical software Stata 14.0.

## Results

### Patient characteristics

**Figure 1.**
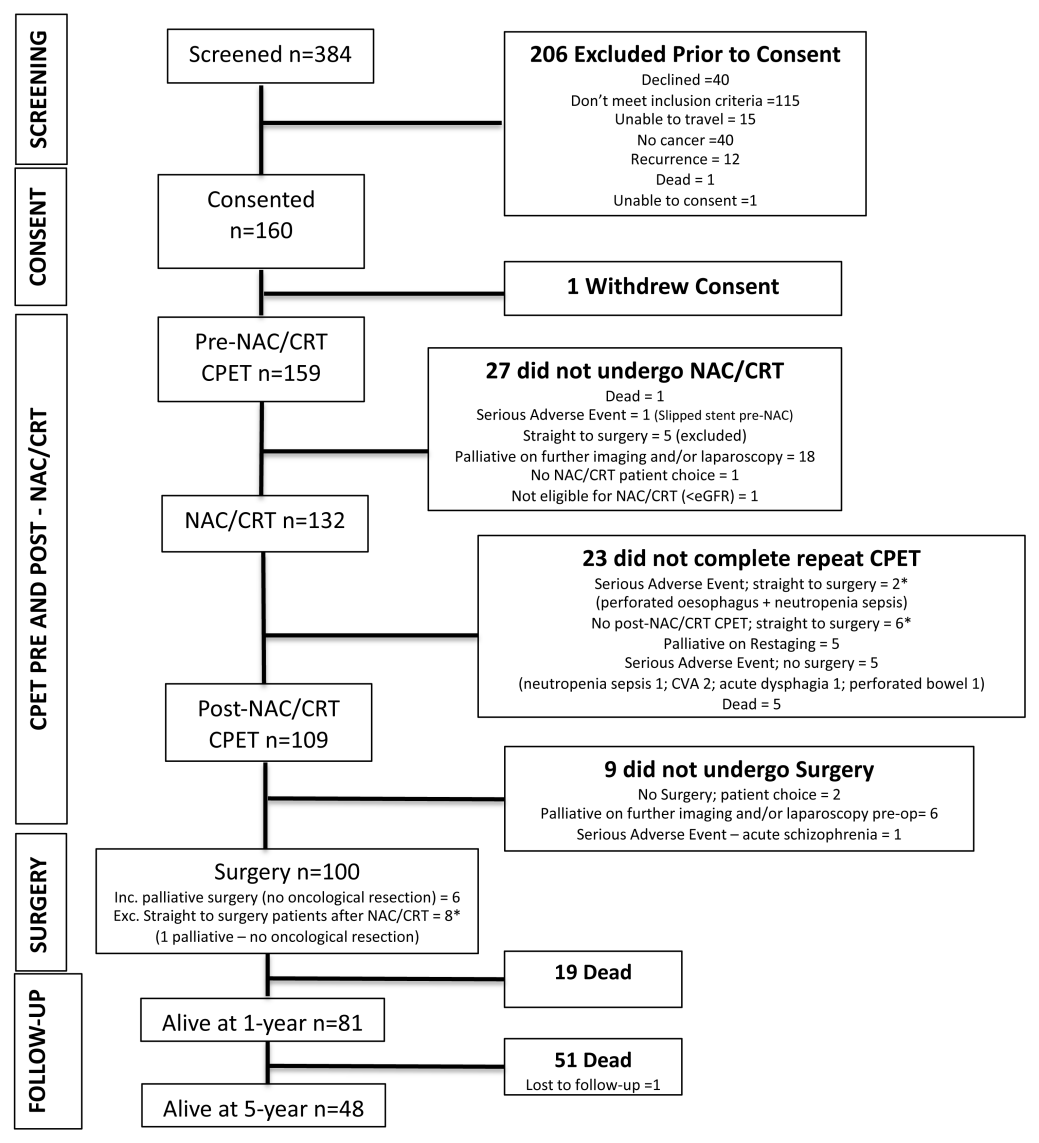
Study enrolment and follow-up. Of the 160 patients who were enrolled, 159 had pre-neoadjuvant chemo/chemoradiotherapy (NAC/CRT) cardiopulmonary exercise testing (CPET), 27 did not undergo NAC/CRT of which 5 went straight to surgery and were excluded. 132 patients underwent NAC/CRT of which 8* went straight to surgery and a further 15 did not complete post-NAC/CRT CPET. 109 patients underwent post-NAC/CRT CPET and 108 underwent surgery. * denotes patients who went straight to surgery after either a serious adverse event during NAC/CRT or patients who did not undergo CPET after NAC/CRT.

From September 2011 to September 2016, we enrolled 160 patients (one-year mortality follow-up to September 2017). One withdrew consent and was not included in the analysis ( Figure 1). Patient characteristics for the whole group are shown in Table 1. Cancer regime, tumour characteristics and radiological responses to chemo/chemoradiotherapy using RECIST v1.1 criteria are presented in Table 2. Patients who completed a pre-chemo/chemoradiotherapy CPET only (i.e. patients who did not progress to surgery due to a serious adverse event, a palliative diagnosis on restaging, death during chemo/chemoradiotherapy or progressing to surgery after a serious adverse event or no CPET; n=23) had lower rates of chemo/chemoradiotherapy completion (7/23 (30.4%) vs. 86/109 (78.9%)), cycles undertaken (1.9 vs. 2.9) and were found to have distant disease on restaging (6/23 (50%) vs. 6/109 (5.5%)). No adverse events during CPET were recorded.

**Table 1.** Characteristics of all recruited patients, including patients that completed neoadjuvant chemotherapy/chemoradiotherapy and cardiopulmonary exercise testing (CPET).

Patient characteristics	All patients (n=159)	Received NAC/CRT (n=132)	Did not receive NAC/CRT (n = 27)	Had repeat CPET (n=109)	Did not have repeat CPET (n=50)
**Male**	114 (71.7%)	99 (75.0%)	15 (56%)	82 (75.2%)	32 (64%)
**Age (years)**	64.6 (9.3)	63.8 (9.2)	68.4 (8.8)	63.5 (9.0)	66.9 (9.7)
**Weight (kg)**	77.68 (17.2)	78.1 (15.8)	75.7 (23.1)	78.5 (15.2)	75.9 (21.0)
**BMI (kg.m ^-2^)**	26.8 (5.2)	26.8 (4.7)	26.8 (7.1)	27.0 (4.6)	26.5 (6.3)
**Smoking**					
Never	42 (26.3%)	31 (23.5%)	11 (41%)	24 (22.0%)	18 (36%)
Previous	86 (54.1%)	75 (56.8%)	11 (41%)	63 (57.8%)	23 (46%)
Current	28 (17.6%)	23 (17.4%)	5 (19%)	19 (17.4%)	9 (18%)
Unknown	3 (1.9%)	3 (2.3%)	0 (0%)	3 (2.8%)	0 (0%)
**Alcohol**					
Never	34 (21.4%)	25 (18.9%)	9 (33%)	21 (19.3%)	13 (26%)
Minimal	51 (32.1%)	43 (32.6%)	8 (30%)	32 (29.4%)	19 (38%)
Moderate	58 (36.5%)	50 (37.9%)	8 (30%)	44 (40.4%)	14 (28%)
Heavy	12 (7.5%)	10 (7.6%)	2 (7%)	8 (7.3%)	4 (8%)
Unknown	4 (2.5%)	4 (3.0%)	0 (0%)	4 (3.7%)	0 (0%)
**Hospital site**					
Southampton	41 (25.8%)	41 (31.1%)	0 (0%)	35 (32.1%)	6 (12%)
Aintree	100 (62.9%)	74 (56.1%)	26 (96%)	59 (54.1%)	41 (82%)
Preston	10 (6.3%)	10 (7.6%)	0 (0%)	10 (9.2%)	0 (0%)
South Tees	8 (5.0%)	7 (5.3%)	1 (4%)	5 (4.6%)	3 (6%)
**Medication**					
Beta-blockers	24 (15.1%)	19 (14.4%)	5 (19%)	16 (14.7%)	8 (16%)
Diabetes medication (oral or insulin)	23 (14.5%)	18 (13.6%)	5 (19%)	15 (13.8%)	8 (16%)
Asthma (inhalers)	18 (11.3%)	15 (11.4%)	3 (11%)	12 (11.0%)	6 (12%)
Antihypertensive	55 (34.6%)	42 (31.8%)	13 (48%)	37 (33.9%)	18 (36%)
Steroids (inhaled)	5 (3.1%)	4 (3.0%)	1 (4%)	4 (3.7%)	1 (2%)
Anticoagulants (Warfarin or Novel Anticoagulants)	4 (2.5%)	4 (3.0%)	0 (0%)	3 (2.8%)	1 (2%)

Data are either n (%) or Mean (SD). Abbreviations: CPET – cardiopulmonary exercise testing; NAC/CRT – neoadjuvant chemotherapy or chemoradiotherapy. Note: All patients (n=159) represent those who underwent baseline assessment; received NAC/CRT (n=132) those who underwent NAC/CRT; did not receive NAC/CRT (n=27) those who did not complete NAC/CRT; had repeat CPET (n=109) those who underwent a pre- and post-NAC/CRT CPET; did not have repeat CPET (n=50) those who did not undertake a post-NAC/CRT CPET. Out of 159 patients, two patients (1%) were diagnosed with a mucinous tumour of the oesophagus (one was palliated), 31 (19%) with squamous-cell carcinoma of the oesophagus (1 oesophagogastric junction) and 126 (79%) patients were diagnosed with adenocarcinoma (oesophageal - 82 patients, gastric – 33 patients and oesophagogastric junction – 11 patients).

**Table 2.** Cancer regime, tumour characteristics and radiological responses of patients who underwent neoadjuvant chemotherapy (NAC) or chemoradiotherapy (CRT).

	Patients completed NAC/CRT (n=132)	CPET Pre- and Post-NAC/CRT (n=109)	CPET Pre- NAC/CRT only (n=23)	P-value
**Cancer therapy regime**				
Chemotherapy (NAC)	113 (85.6%)	93 (85.3%)	20 (87.0%)	1.0
Chemoradiotherapy (CRT)	19 (14.4%)	16 (14.7%)	3 (13.0%)	
**Cancer therapy regime**				
Complete	93 (70.5%)	86 (78.9%)	7 (30.4%)	**0.0015**
Incomplete	39 (29.5%)	23 (21.1%)	16 (69.6%)	
**Number of Cycles**	2.8 (0.9)	2.9 (0.9)	1.9 (0.7)	**0.0012**
**Pre-NAC/CRT Radiological Staging**				
Tumour stage (T-stage) *				
T2	41 (31.1%)	32 (29.4%)	9 (39.1%)	0.826
T3	81 (61.4%)	68 (62.4%)	13 (56.5%)	
T4	10 (7.6%)	9 (8.3%)	1 (4.4%)	
Nodal stage (N-Stage) *				
N0	19 (14.4%)	14 (12.8%)	5 (21.7%)	0.276
N1	85 (64.4%)	71 (65.1%)	14 (60.9%)	
N2	26 (19.7%)	23 (21.1%)	3 (13.0%)	
N3	2 (1.5%)	1 (0.9%)	1 (4.4%)	
**Post-NAC/CRT Radiological Staging ^a^**				
Tumour stage (T-stage) *				
T0	11 (9.1%)	11 (10.1%)	0 (0%)	0.349
T1	6 (5.0%)	6 (5.5%)	0 (0%)	
T2	28 (23.1%)	26 (23.9%)	2 (16.7%)	
T3	69 (57.0%)	61 (56.0%)	8 (66.7%)	
T4	7 (5.8%)	5 (4.6%)	2 (16.7%)	
Nodal Stage (N-stage) *				
N0	51 (42.1%)	47 (43.1%)	4 (33.3%)	0.089
N1	39 (32.2%)	37 (33.9%)	2 (16.7%)	
N2	29 (24.0%)	24 (22.0%)	5 (41.7%)	
N3	2 (1.7%)	1 (0.9%)	1 (8.3%)	
Metastasis (M-stage) *				
M1	12 (9.9%)	6 (5.5%)	6 (50%)	**0.0010**
**Tumour type**				
Adenocarcinoma	106 (80.3%)	90 (82.6%)	16 (69.6%)	0.279
Squamous-cell carcinoma	25 (18.9%)	18 (16.5%)	7 (30.4%)	
Other	1 (0.8%)	1 (0.9%)	0 (0%)	
**NAC/CRT radiological response ^b$^**				
Stable disease	57 (44.2%)	49 (45.0%)	8 (40.0%)	**0.0003**
Partial response	63 (48.8%)	59 (54.1%)	4 (20.0%)	
Unknown	9 (7.0%)	1 (0.9%)	8 (40.0%)	

Data are n (%) or mean (SD) unless otherwise stated. ^a^ n=121, ^b^ n=129. ^$^Radiological responses post-NAC/CRT were based on definitions outlined by the RECIST version 1.1 criteria for solid target lesions. P-value for comparison between pre- and post NAC group vs. pre-NAC group. Statistical significance was taken <0.05 and highlighted in bold. Note: Complete cancer therapy is defined as successfully completion of all planned cycles of NAC/CRT. Incomplete cancer therapy is defined as unsuccessful completion of all planned NAC/CRT cycles for any reason. * T-stage, N-stage, M-stage both pre and post-NAC/CRT was assessed by means of computer tomography (CT) and/or endoscopic ultrasound and/or ^18^F-fluorodeoxyglucose positron-emission tomography, classified according to TNM v7.

### Change in physical fitness following chemo/chemoradiotherapy

**Figure 2.**
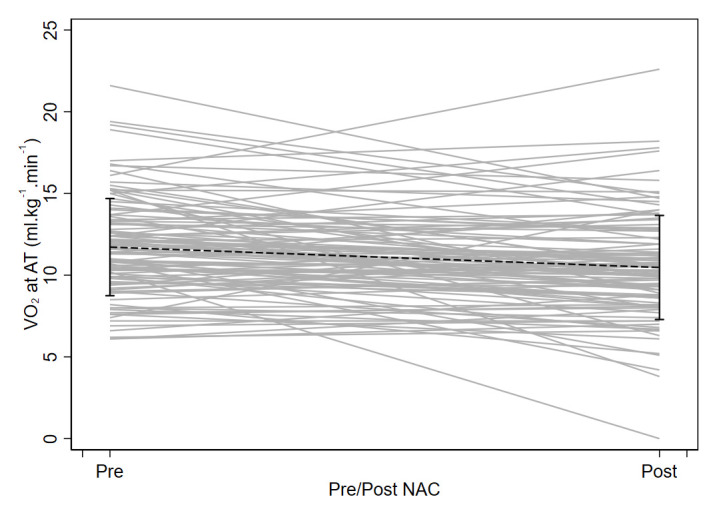
A ladder plot of oxygen uptake at anaerobic threshold (VO _2_ at AT in ml.kg ^−1^.min ^−1^) pre (1) and post (2) chemo/chemoradiotherapy (NAC/CRT).

Table 3 summarises CPET data for patients who completed chemo/chemoradiotherapy. There was a significant decline in VO _2_ at AT and VO _2_ at Peak: -1.25ml.kg ^-1^.min ^-1^ (-1.80 to -0.69) and -3.02ml.kg ^-1^.min ^-1^ (-3.85 to -2.20); p<0.0001) following neoadjuvant chemo/chemoradiotherapy. Other key CPET variables are summarised in Table 3, all showing a significant reduction in fitness. Patients whose treatment pathway changed, i.e. did not complete chemo/chemoradiotherapy (n=50), were found to be significantly more unfit on their baseline CPET compared to patients who completed chemo/chemoradiotherapy (VO _2_ at Peak 18.6 (5.7) ml.kg ^-1^.min ^-1^ vs. 20.8 (5.9) ml.kg ^-1^.min ^-1^: p=0.025 and work rate at peak 104.8 (47.9)W vs. 134.8 (49.6)W; p<0.001. Figure 2 demonstrates a graphical representation of the VO _2_ at AT data pre- and post-chemo/chemoradiotherapy. Based on our predetermined risk stratification thresholds, 47% of patients changed fitness group following chemo/chemoradiotherapy (51/109), of these 10% moved to a lower risk group, i.e. improved their fitness (11/109) and 37% moved to a higher risk group (40/109), with no change in 53% (58/109). 

**Table 3.** Cardiopulmonary exercise testing (CPET) variables before and after neoadjuvant chemotherapy (NAC) and chemoradiotherapy (CRT).

	Pre-NAC/CRT (n=109)	Post-CRT/NAC (n=109)	Difference (95% CI)	P-value
**VO _2_ at AT (ml.min ^-1^)**	0.91 (0.27)	0.8 (0.26)	-0.11 (-0.16 to -0.07)	**<0.0001**
**VO _2_ at AT (ml.kg ^-1^.min ^-1^)**	11.72 (2.97)	10.47 (3.18)	-1.25 (-1.80 to -0.69)	**<0.0001**
**VO _2_ at Peak (ml.min ^-1^)**	1.62 (0.51)	1.37 (0.49)	-0.25 (-0.32 to -0.18)	**<0.0001**
**VO _2_ at Peak (ml.kg ^-1^.min ^-1^)**	20.81 (5.93)	17.79 (5.58)	-3.02 (-3.85 to -2.20)	**<0.0001**
**WR at AT (W)**	62.9 (30.76)	53.19 (28.35)	-9.71 (-14.37 to -5.04)	**0.0001**
**WR at Peak (W)**	134.8 (49.6)	117.39 (48.29)	-17.39 (-24.08 to -10.69)	**<0.0001**
**V _E_/VCO _2_ at AT**	35.11 (4.50)	36.96 (5.93)	1.85 (0.94 to 2.76)	**<0.0001**
**V _E_/VCO _2_ at Peak**	35.89 (4.43)	37.61 (5.73)	1.73 (0.96 to 2.49)	**<0.0001**
**V _E_/VO _2_ at AT**	29.49 (4.44)	32.65 (6.46)	3.15 (2.10 to 4.21)	**<0.0001**
**V _E_/VO _2_ at Peak**	41.86 (7.40)	45.73 (10.14)	3.87 (2.50 to 5.25)	**<0.0001**
**PETCO _2_ at AT**	37.52 (3.20)	35.95 (5.07)	-1.57 (-2.48 to -0.66)	**0.0009**
**PETCO _2_ at Peak**	35.39 (3.64)	34.18 (4.17)	-1.20 (-1.77 to -0.63)	**0.0001**
**VO _2_/HR at AT (ml.beat ^-1^)**	8.84 (2.68)	7.70 (2.31)	-1.13 (-1.48 to -0.79)	**<0.0001**
**VO _2_/HR at Peak (ml.beat ^-1^)**	11.47 (3.27)	9.98 (3.12)	-1.49 (-1.87 to -1.11)	**<0.0001**

Data are mean (SD) unless otherwise stated. Statistical significance was taken as <0.05 and highlighted in bold. Abbreviations: NAC (neoadjuvant chemotherapy), CRT (neoadjuvant chemoradiotherapy), VO _2 _at AT (oxygen uptake at anaerobic threshold), VO _2 _at Peak (oxygen uptake at peak exercise), WR at AT (work rate at anaerobic threshold), WR at Peak (work rate at peak exercise), V _E_/VCO _2 _at AT (ventilatory equivalent for carbon dioxide at the anaerobic threshold), V _E_/VCO _2 _at Peak (ventilatory equivalent for carbon dioxide at peak exercise), V _E_/VO _2 _at AT (ventilatory equivalent for oxygen at the anaerobic threshold), V _E_/VO _2 _at Peak (ventilatory equivalent for oxygen at peak exercise), PETCO _2 _at AT (end tidal carbon dioxide at the anaerobic threshold), PETCO _2 _at Peak (end tidal carbon dioxide at peak exercise), VO _2_/HR at AT (oxygen pulse at the anaerobic threshold), VO _2_/HR at Peak (oxygen pulse at peak exercise

### Fitness and survival

**Figure 3.**
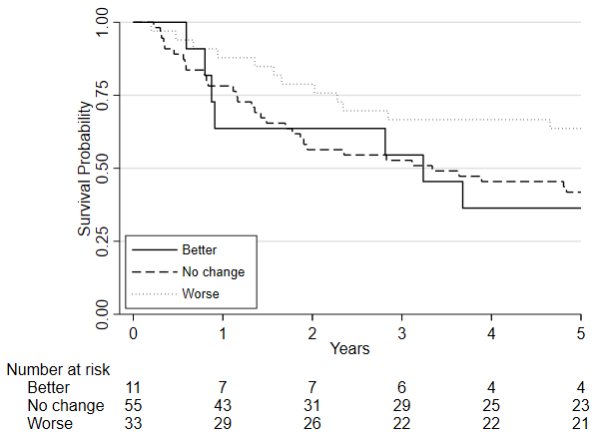
Kaplan-Meier plot of the estimated overall 5-year survival among patients with oesophago-gastric cancer who underwent CPET pre-and post-NAC/CRT followed by surgery, based on their change in VO _2_ at AT risk stratification category following chemo/chemoradiotherapy (NAC/CRT).

**Figure 4.**
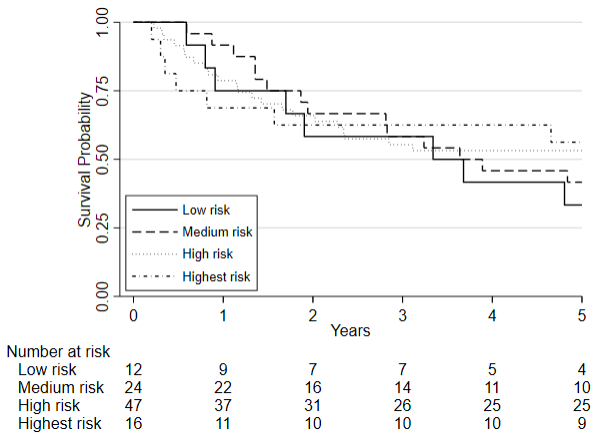
Kaplan-Meier plot of the estimated overall 5-year survival among patients with oesophago-gastric cancer who underwent CPET pre-and post-NAC/CRT followed by surgery, based on their post-chemo/chemoradiotherapy (NAC/CRT) VO _2_ at AT risk stratification category. Predefined risk stratification categories were defined as low risk VO _2_ at AT >14ml.kg. ^-1^min ^-1^, medium risk VO _2_ at AT 11.0-14.0ml.kg ^-1^.min ^-1^, high-risk VO _2_ at AT 8.0-10.9ml.kg ^-1^.min ^-1^, highest risk VO _2_ at AT <8.0ml.kg ^-1^.min ^-1^.

**Figure 5.**
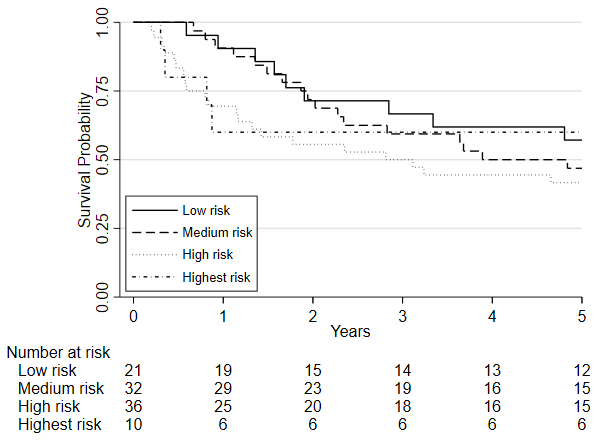
Kaplan-Meier plot of the estimated overall 5-year survival among patients with oesophago-gastric cancer who underwent CPET pre-and post-NAC/CRT followed by surgery, based on their pre-chemo/chemoradiotherapy (NAC/CRT) VO _2_ at AT risk stratification category. Predefined risk stratification categories were defined as low risk VO _2_ at AT >14ml.kg. ^-1^min ^-1^, medium risk VO _2_ at AT 11.0-14.0ml.kg ^-1^.min ^-1^, high-risk VO _2_ at AT 8.0-10.9ml.kg ^-1^.min ^-1^, highest risk VO _2_ at AT <8.0ml.kg ^-1^.min ^-1^.

Survival analyses was based on 100 patients (19 of whom died) who had repeat CPET following chemo/chemoradiotherapy, underwent surgery and were followed one year later. Survival analyses was also conducted on 99 patients five years later (51 of whom died). There was insufficient evidence that a change in fitness between pre- and post-chemo/chemoradiotherapy (HR=0.88, 95%CI: 0.75 to 1.03, p=0.115) was independently associated with 1-year mortality whilst there was a significant change associated with 5-year mortality (HR = 0.89 (0.81 to 0.98; p = 0.019); Figure 3). There was also insufficient evidence that post-chemo/chemoradiotherapy fitness was independently associated with one-year mortality (HR = 0.90, 95%CI: 0.78 to 1.03, p=0.122) and 5-year mortality (HR = 1.06 (0.98 to 1.15; p = 0.135); Figure 4). Pre-chemo/chemoradiotherapy (baseline) VO _2_ at AT was however independently associated with 1-year mortality (HR=0.72, 95%CI: 0.59 to 0.88, p=0.001) whilst there was insufficient evidence for 5-year mortality (HR = 0.97 (0.88 to 1.06; p = 0.494); Figure 5).

**Figure 6.**
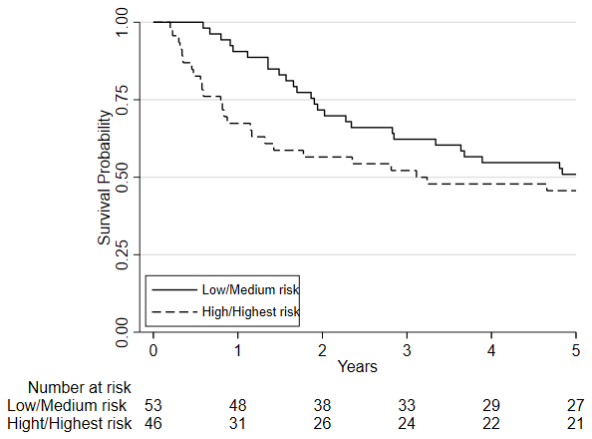
A Kaplan-Meier plot of the estimated overall 5-year survival estimates among patients with oesophageal, esophagogastric-junction and gastric cancer who underwent CPET pre-and post-chemotherapy or chemoradiotherapy (NAC/CRT) followed by surgery. Patients are split based on their pre-NAC/CRT VO2 at AT using predefined risk stratification categories (low risk Vo2 at AT >14ml.kg.-1min-1, medium risk Vo2 at AT 11.0-14.0ml.kg-1.min-1, high-risk Vo2 at AT 8.0-10.9ml.kg-1.min-1, highest risk Vo2 at AT <8.0ml.kg-1.min-1).

**Figure 7.**
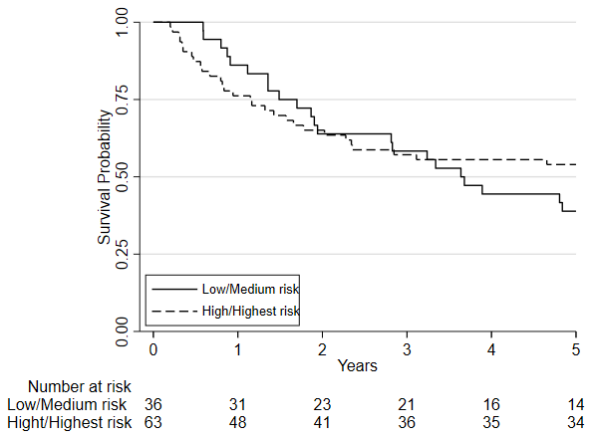
Kaplan-Meier plot of the estimated overall 5-year survival estimates among patients with oesophageal, esophagogastric-junction and gastric cancer who underwent CPET pre-and post-chemotherapy or chemoradiotherapy (NAC/CRT) followed by surgery. Patients are split based on their post-NAC/CRT VO2 at AT using predefined risk stratification categories (low risk VO2 at AT >14ml.kg.-1min-1, medium risk VO2 at AT 11.0-14.0ml.kg-1.min-1, high-risk VO2 at AT 8.0-10.9ml.kg-1.min-1, highest risk VO2 at AT <8.0ml.kg-1.min-1).

Considering clinical risk groups, pre-chemo/chemoradiotherapy VO _2_ at AT risk group (low/medium risk vs. high/highest risk) was associated with mortality at one-year (HR=7.06; 95%CI: 2.04 to 24.40; p=0.002) whilst there was insufficient evidence for mortality at five-year follow (HR = 1.39 (0.80 to 2.41; p=0.237)) ( Figure 6). Post-chemo/chemoradiotherapy showed weaker evidence for an association at one year (HR=3.29; 95%CI: 0.95 to 11.36; p=0.06) and there was insufficient evidence for five-year mortality (HR = 0.79 (0.46 to 1.39; p = 0.426) ( Figure 7). A weak relationship between change in fitness risk group and survival at one year was noted (Better HR=0.78, 95%CI: 0.17 to 3.48; Worse HR=0.53, 95%CI: 0.17 to 1.64; p=0.550) whilst a stronger relationship was noted at 5 years (Better HR = 1.09, 95%CI: 0.48 to 2.48; Worse HR = 0.52, 95%CI: 0.27 to 1.01; p=0.097).

Pre-chemo/chemoradiotherapy VO _2_ at Peak was also independently associated with 1-year mortality (HR=0.85, 95%CI: 0.76 to 0.95, p=0.005). There was no evidence of an association between VO _2_ at Peak post-chemo/chemoradiotherapy or relative change in VO _2_ at Peak from pre- to post-chemo/chemoradiotherapy. Following adjustment for post-chemo/chemoradiotherapy VO _2_ at Peak, the relative change in VO _2_ at Peak following chemo/chemoradiotherapy was associated with 1-year mortality (HR=0.85, 95%CI: 0.74 to 0.98, p=0.023). 

### Fitness and post-operative outcomes

Of the 109 patients who complete chemo/chemoradiotherapy, 108 underwent surgery (100 patients had complete pre- and post-chemo/chemoradiotherapy CPET data as well as surgical outcomes). Surgery and histopathological outcomes are summarised in Table 4. A total 6% of patients underwent a palliative bypass, 57% of patients underwent a laparoscopic operation with only one patient having a conversion to open surgery. An R0 resection was achieved in 75% of patients. A complete pathological response (TRG1) was achieved in 14%. Table 5 summarizes the post-operative outcomes as defined by POMS at Day3, 5, 8 and 15. Of note, on Day 8, 24% of patients had a complication in the low-risk group, whilst 46% of patients still had a complication in the highest risk. Median length of stay was 12 days for the low-risk group and 13.5 days in the highest risk group. Seven patients in the low/medium risk groups died post-operatively at 1-year (11.6%) whilst 18 patients died in the high/highest risk groups (36.7%). Thirty-one patients in the low/medium risk groups died post-operatively at five-years (51.6 %) whilst 28 patients died in the high/highest risk groups (57.1 %). There was insufficient evidence of an association found between POMS at Day 3, 5, 8 and 15 and pre- chemo/chemoradiotherapy VO _2_ at AT (OR=1.04 95%CI: 0.85 to 1.27; p=0.730). Further, insufficient evidence of relationships was found with either post-chemo/chemoradiotherapy fitness or change in fitness. Similar results were seen for VO _2_ at Peak. Of note 46% of highest risk patients were discharged back to their own home post-operatively (compared with 95% in the low-risk group), and 46% of patients in the high-risk group were re-admitted within 30-days (compared with 5% in the low-risk group).

**Table 4.** Surgery and Histopathological Outcomes.

	Pre-and post- NAC/CRT data and surgery (n=100)	Pre-NAC/CRT data and surgery (n=8)	P-value
**Operation**			
Esophagectomy	71 (71%)	4 (50.0%)	0.324
Gastrectomy	23 (23%)	3 (37.5%)	
Palliative	6 (6%)	1 (12.5%)	
**Operation type**			
Unknown	0 (0%)	0 (0%)	0.605
Open	35 (35%)	3 (37.5%)	
Laparoscopic	58 (58%)	4 (50%)	
Laparoscopic converted to open	1 (1%)	0 (0%)	
Palliative resection	6 (6%)	1 (12.5%)	
**Pathology staging**			
Tumour stage (T-stage) ^a*^			
0	16 (15.0%)	0 (0%)	0.459
1a +1b	11 (11.7%)	1 (14.3%)	
2 +2a	14 (14.9%)	2 (28.6%)	
3	47 (50.0%)	3 (42.9%)	
4	6 (6.4%)	1 (14.3%)	
Nodal stage (N-Stage) ^a*^			
0	43 (45.7%)	4 (57.1%)	1
1	25 (26.6%)	2 (28.6%)	
2	18 (19.2%)	1 (14.3%)	
3	5 (5.3%)	0 (0%)	
3a	3 (3.2%)	0 (0%)	
**Resection (R0/R1) ^a^**			
0	75 (79.8%)	6 (85.7%)	1
1	19 (20.2%)	1 (14.3%)	
**Tumour Regression Grade ^b^**			
1	15 (16.0%)	0 (0%)	0.579
2	10 (10.6%)	1 (16.7%)	
3	16 (17.0%)	2 (33.3%)	
4	23 (24.5%)	2 (33.3%)	
5	30 (31.9%)	1 (16.7%)	
**EMVI (yes) ^a^**	28 (29.8%)	1 (14.3%)	0.670
**Differentiation ^c^**			
Moderate	35 (36.8%)	2 (28.6%)	0.695
None	13 (13.7%)	0 (0%)	
Poor	42 (44.2%)	5 (71.4%)	
Well	5 (5.3%)	0 (0%)	

Data are n (%) unless otherwise stated. Statistical significance was taken as <0.05 and highlighted in bold. Abbreviations: NAC (neoadjuvant chemotherapy), CRT (neoadjuvant chemoradiotherapy. * T-stage and N-stage classified according to TNM v7. ^a ^n=101, ^b ^n=100, ^c ^n=102. ^$ ^Tumor Regression Grade (TRG) was based on definitions outlined by the Mandard score. EMVI – Extra mural venous invasion. RO – complete resection with no tumor within 1mm of the resection margin.

**Table 5.** Post-operative outcomes for low, medium, high and highest risk groups categorised by pre-chemotherapy/chemoradiotherapy oxygen uptake at anaerobic threshold (ml.kg ^-1^.min ^-1^).

	Low (n=21)	Medium (n=39)	High (n=38)	Highest (n=11)
**Post-Operative Morbidity Survey (POMS)**				
Day 3 ^a^	20 (95.2%)	31 (79.5%)	33 (86.8%)	10 (90.9%)
Day 5 ^b^	20 (95.2%)	30 (76.9%)	26 (68.4%)	7 (63.6%)
Day 8 ^c^	5 (23.8%)	21 (53.8%)	20 (52.6%)	5 (45.5%)
Day 15 ^d^	4 (19.0%)	7 (17.9%)	5 (13.2%)	1 (9.1%)
**Length of Stay (days) ^a^**	12 (9-15)	13 (10-18)	11 (9-14.5)	13.5 (7-16)
**O-POSSUM Physiology score ^a^**	16.4 (3.3)	17.0 (3.4)	17.7 (2.9)	17.4 (2.8)
**O-POSSUM Mortality score ^a^**	7.8 (4.9)	10.0 (6.0)	12.6 (6.7)	11.5 (5.0)
**RCRI (total score) ^a^**	1.0 (0.2)	1.1 (0.4)	1.2 (0.5)	1.5 (0.8)
**Level 2/3 Length of Stay ^a^**	4 (3-7)	4 (3-5)	4 (2-6)	4 (3-5)
**Discharge Destination**				
Home	20 (95.2%)	39 (100%)	30 (78.9%)	5 (45.5%)
Intermediate care	0 (0%)	0 (0%)	5(13.1%)	3 (9.1%)
Nursing home	0 (0%)	0 (0%)	3 (7.9%)	3 (9.1%)
Rehabilitation hospital	1 (4.8%)	0 (0%)	0 (0%)	0 (0%)
**30-day readmission ^d^**	1 (4.8%)	5 (12.8%)	6 (15.7%)	5 (45.5%)
**1-year Mortality**	2 (9.5%)	5 (12.8%)	14 (36.8%)	4 (36.4%)
**5-year Mortality**	9 (42.9%)	24 (61.5%)	23 (60.5%)	5 (45.4%)

Data are either n (%), mean (SD) or median (IQR) unless otherwise stated. Abbreviations: O-POSSUM – Oesophago-gastric specific Physiological and Operative Severity Score for the enumeration of Mortality and morbidity. ^a^ n=100, ^b^ n=80, ^c^ n=26, ^d ^n=102 Note: VO _2_ at AT (ml.kg. ^-1^min ^-1^) predefined risk stratification categories (low risk VO _2_ at AT >14ml.kg. ^-1^min ^-1^, medium risk VO _2_ at AT 11.0-14.0ml.kg ^-1^.min ^-1^, high-risk VO _2_ at AT 8.0-10.9ml.kg ^-1^.min ^-1^, highest risk VO _2_ at AT <8.0ml.kg ^-1^.min ^-1 ^).

### Fitness and quality of life

We found no evidence of an association between changes in EQ-5D-5L and the relative change in VO _2_ at AT or VO _2_ peak following chemo/chemoradiotherapy at 30-days and one-year post-operatively (Spearman correlation 30-days r=0.062, p=0.611 and r=0.10, p=0.393; 1-year r=0.162, p=0.191 and r=0.141, p=0.253 respectively). 

### Chemo/chemoradiotherapy adverse effects and tolerability

A patient’s ability to complete the planned full cycles of chemo/chemoradiotherapy (tolerability) as a function of their baseline fitness was assessed. Adverse events encountered during chemotherapy and chemoradiotherapy are presented in Table 6.

**Table 6.** Adverse events during chemotherapy and chemoradiotherapy – events of grade ≥2 during chemotherapy or chemoradiotherapy.

Regime	Adverse Events: Number of events/ total number of patients	Adverse Event Description
Epirubicin, Oxaliplatin, Capecitabine (EOX)	0/15	
Epirubicin, Cisplatin, Capecitabine (ECX)	18/71	Neutropenia– 3 Acute kidney injury – 9 Proximal lower limb deep vein thrombosis – 2 Pulmonary embolism – 1 Cerebrovascular accident - 2 Dysphagia and stent placement – 1 Death – 2
Epirubicin, Cisplatin, 5-Fluorouracil (ECF)	1/3	Neutropenia– 1
STO3 trial – ECX alone	1/4	Pulmonary embolism – 1
STO3 trial –ECX + Bevacizumab	1/4	Oesophageal perforation - 1
OEO5 trial – ECX alone	1/2	Death - 1
OEO5 trial – Cisplatin and 5-Fluorouracil	0/6	
CROSS trial – Carboplatin, Paclitaxel with concurrent radiotherapy	0/10	
NEOSCOPE trial – Oxaliplatin and Capecitabine with concurrent radiotherapy	1/6	Death - 1
NEOSCOPE trial –Carboplatin and Paclitaxel with concurrent radiotherapy	0/3	
Herceptin, Cisplatin and Capecitabine	0/2	
Capecitabine alone	0/1	
Cisplatin alone	4/5	Death – 1 Neutropenia – 1 Acute kidney injury– 1 Acute psychosis - 1

Adverse events were graded according to the National Cancer Institute’s Common Terminology Criteria for Adverse Events, version 3.0. Neutropenia is defined as absolute neutrophil count <1.0 ×10 ^9^/L; Creatinine >1.5 upper limit for normal Of 132 patients receiving NAC/CRT; 86% underwent chemotherapy alone (38 patients (34%) incomplete treatment) and 14% underwent chemoradiotherapy (1 patient (5%) incomplete treatment). Acute kidney injury with a rise in serum creatinine more than 1.5 times the upper limit of normal was the most common adverse event encountered. Five deaths were recorded during NAC/CRT.

No association between VO _2_ at AT or VO _2_ peak and tolerability was found (p=0.161 and p=0.057). One-year survival was no better for patients who tolerated chemo/chemoradiotherapy (HR = 0.42, 95%CI: 0.17 to 1.06; p=0.067). Additionally, there was no evidence that either morbidity at day 5 or EQ-5D at 30-days post-operatively was associated with tolerance (p=0.149 and p=0.132).

### Change in fitness with chemotherapy vs. chemoradiotherapy

A substantial clinically important significant difference in VO _2_ at AT was observed between patients undergoing chemoradiotherapy vs. chemotherapy groups (-2.7(3.7) ml.kg ^-1^.min ^-1 ^vs. -0.9(2.8) ml.kg ^-1^.min ^-1^; p=0.025). However, a non-significant difference in VO _2_ at Peak, was observed between both groups ( -3.8(5.7) ml.kg ^-1^.min ^-1 ^vs. -2.8(4.2) ml.kg ^-1^.min ^-1^; p=0.380).

## Discussion

This prospective, observer blinded, multi-centre, observational, cohort study in patients undergoing multimodal neoadjuvant cancer therapies for locally advanced OG cancers showed a significant decline in objectively measured fitness (VO _2_ at AT and VO _2_ peak) following chemo/chemoradiotherapy. Importantly, baseline fitness (VO _2_ at AT and VO _2 _peak) was strongly associated with mortality at one-year. Finally, we observed a higher incidence of post-operative morbidity and a longer length of hospital stay in the high/highest risk groups, as well as a greater decline in VO _2_ at AT following chemoradiotherapy compared to chemotherapy treatment.

Multimodal neoadjuvant therapies for OG cancer patients are associated with marginal gains in overall survival ^6, 31^. However, these treatments might be accompanied with a significant cost to patient’s fitness. Although overall survival has improved for oesophageal patients, a network meta-analyses showed that chemoradiotherapy increased the risk of post-operative mortality when compared to chemotherapy or surgery alone, but improved tumour regression was found ^32^. Similarly, overall survival was moderately improved in gastric cancer patients undergoing platinum based triplet regimens ^6, 33^, however such patients experience significant peri-chemotherapy morbidity with increased lymphocytopenia and hemoglobinopathy. In some patients, pre-operative treatment might have no meaningful benefit and may even cause harm ^34^.

The reliability and association of selected CPET variables with post-operative outcome has been established for major abdominal surgery ^9^, however this relationship has not been adequately investigated in OG patients, where surgery is frequently in two body cavities. At present, there is little evidence supporting the use of pre-operative CPET to aid shared decision making and guide perioperative care prior to OG cancer surgery. Conclusive evidence regarding objective changes in fitness after neoadjuvant treatment for OG cancer and any possible relationship with operative outcomes does not exist. So far small, unblinded, single-centre, observational studies report similar significant declines in fitness with chemo/chemoradiotherapy, associations between low fitness and higher post-operative cardio-respiratory complications and sustained reductions in fitness between the end of neoadjuvant chemotherapy (NAC) and surgery ^10– 13^.

This study reports clinically important reductions in fitness associated with chemo/chemoradiotherapy, validating our initial pilot and single-centre observations ^10– 13^. This may be attributed to changes in metabolic health such as changes in body composition (sarco-cachexia) due to neoadjuvant treatments, recently recognised in OG cancer cohorts ^35– 37^. Sarcopenia and sarcopenic obesity are associated with early termination of neoadjuvant treatments, dose limiting toxicity, operative morbidity, poor oncological outcomes, including poor survival ^38^. Sarcopenia, like poor fitness is a modifiable risk factor. Multimodal prehabilitation interventions might rescue the decline in fitness and body composition seen with neoadjuvant treatments ^39– 41^, however, improving baseline fitness (associated with one-year mortality) is challenging and a broader public health issue. Whilst mechanisms of reduced fitness are unclear, a reduction in muscle mass is also known to reduce ventilatory efficiency. This study demonstrates a reduction in ventilatory equivalent for carbon dioxide (V _E_/VCO _2_) at AT and peak. Moreover, mitochondrial dysfunction and sarcopenia are attributed to toxicity from neoadjuvant platinum-based compounds. Mitochondrial DNA, cell cycle arrest ^42^, sustained activation of degradative proteasome and autophagy systems ^43^, and altered NF-kB signalling ^44^ are linked to platinum based chemotherapy toxicity. Ultimately, these changes induce mitochondrial and cellular protein damage, leading to autologous destruction – mytophagy and muscle wasting ^45^ seen in the present study as a reduction in oxygen utilisation and power output at AT and peak exercise.

Objective fitness assessment prior to chemo/chemoradiotherapy provides useful information to guide personalized shared decision-making around operative risk and survival for the multi-disciplinary team and the patient. It may also guide neoadjuvant chemo/chemoradiotherapy choices, by directing more aggressive chemo/chemoradiotherapy choices to fitter individuals or even guide delays in the cancer treatment pathway to institute an intervention before neoadjuvant treatment in selected individuals. Informing the selection of risk-reducing neoadjuvant treatment options when baseline fitness is already compromised (e.g. selection of NAC over chemoradiotherapy for low baseline fitness patients), or even guide shared-decision making around palliative options might be clinically desirable. On the contrary, giving higher doses of chemotherapy or chemoradiation, or even more aggressive FLOT-based chemotherapy ^32^ in patients who are objectively in low-risk fitness categories might improve outcomes for selected patients. Objective risk-stratification might also direct higher levels of peri/post-operative care in unfit individuals, thereby improve utilisation of scarce critical care resources. The more information we are able to obtain on the patient’s physiology, metabolic health and tumour status, the more we are able to effectively inform patients and their relatives in their decision-making process and consent. These data might also suggest the potential utility of tailored prehabilitation interventions during neoadjuvant treatments to optimise metabolic health and respiratory function, however this needs further evaluation as maintaining/improving fitness during the whole cancer pathway might impart some long-term benefits ^40, 46, 47^.

There are several strengths to this study. Firstly, it validates the findings of other smaller cohort studies utilising rigorous, prospective, observer-blinded methodology and providing multi-centre generalisability. It describes a novel, strongly independent association between baseline physical fitness (pre- chemo/chemoradiotherapy) and one-year overall survival, but unfortunately not between post-treatment fitness and survival. These observations emphasise the need for physiological staging, including evaluation of physical fitness, to be undertaken prior to chemo/chemoradiotherapy, and that fitness assessment after chemo/chemoradiotherapy might not play a role in predicting long-term outcomes. Further work is needed to establish this. Methodological rigor around dual-expert, blind CPET reporting and a low risk of confounding by indication is also a strength.

Limitations of this study included, a lack of accurate operative definitions, the heterogeneity of the cancer type and cancer treatments. This, however, is a pragmatic study and restrictions based around operative technique and care treatment were felt to be overly restrictive and not generalisable. Further, inability to successfully interrogate the association between fitness, morbidity and patient reported quality of life was due to a lack of statistical power. Furthermore, neither changes in physical fitness nor fitness-risk group following chemo/chemoradiotherapy were independently associated with one-year survival due to a lack of statistical power.

In conclusion, neoadjuvant treatment prior to OG cancer surgery significantly reduces physical fitness, with patients who are unfit at baseline having lower survival at one-year post-operatively.

## Data availability

### Underlying data

Harvard Dataverse: Replication Data for Demographics: The effects of cancer therapies on physical fitness before oesophagogastric cancer surgery: A prospective, blinded, multi-centre, observational, cohort study’. https://doi.org/10.7910/DVN/ILSTAD ^48^.

Harvard Dataverse: Replication Data for EQ-5D-5L Pre NAC: The effects of cancer therapies on physical fitness before oesophagogastric cancer surgery: A prospective, blinded, multi-centre, observational, cohort study’. https://doi.org/10.7910/DVN/Z0H41K ^49^.

Harvard Dataverse: Replication Data for EQ-5D-5L Post NAC: The effects of cancer therapies on physical fitness before oesophagogastric cancer surgery: A prospective, blinded, multi-centre, observational, cohort study’. https://doi.org/10.7910/DVN/RNVIHN ^50^.

Harvard Dataverse: Replication Data for RCRI: The effects of cancer therapies on physical fitness before oesophagogastric cancer surgery: A prospective, blinded, multi-centre, observational, cohort study’. https://doi.org/10.7910/DVN/GQTBTM ^51^.

Harvard Dataverse: Replication Data for O-POSSUM: The effects of cancer therapies on physical fitness before oesophagogastric cancer surgery: A prospective, blinded, multi-centre, observational, cohort study’. https://doi.org/10.7910/DVN/XSJHSZ ^52^.

Harvard Dataverse: Replication Data for POMS Day 3: The effects of cancer therapies on physical fitness before oesophagogastric cancer surgery: A prospective, blinded, multi-centre, observational, cohort study’. https://doi.org/10.7910/DVN/GTE1QJ ^53^.

Harvard Dataverse: Replication Data for POMS Day 5: The effects of cancer therapies on physical fitness before oesophagogastric cancer surgery: A prospective, blinded, multi-centre, observational, cohort study’. https://doi.org/10.7910/DVN/XIXD2L ^54^.

Harvard Dataverse: Replication Data for POMS Day 15: The effects of cancer therapies on physical fitness before oesophagogastric cancer surgery: A prospective, blinded, multi-centre, observational, cohort study’. https://doi.org/10.7910/DVN/BAHW0O ^55^.

Harvard Dataverse: Replication Data for Op Details: The effects of cancer therapies on physical fitness before oesophagogastric cancer surgery: A prospective, blinded, multi-centre, observational, cohort study’. https://doi.org/10.7910/DVN/LOB8PG ^56^.

Harvard Dataverse: Replication Data for Histology: The effects of cancer therapies on physical fitness before oesophagogastric cancer surgery: A prospective, blinded, multi-centre, observational, cohort study’. https://doi.org/10.7910/DVN/DSCSOI ^57^.

Harvard Dataverse: Replication Data for EQ-5D-5L 30 days post surgery: The effects of cancer therapies on physical fitness before oesophagogastric cancer surgery: A prospective, blinded, multi-centre, observational, cohort study’. https://doi.org/10.7910/DVN/ZDR7C1 ^58^.

Harvard Dataverse: Replication Data for EQ-5D-5L 1 year post surgery: The effects of cancer therapies on physical fitness before oesophagogastric cancer surgery: A prospective, blinded, multi-centre, observational, cohort study’. https://doi.org/10.7910/DVN/AGC3IL ^59^.

Harvard Dataverse: Replication Data for CPET Variables: The effects of cancer therapies on physical fitness before oesophagogastric cancer surgery: A prospective, blinded, multi-centre, observational, cohort study’. https://doi.org/10.7910/DVN/IM11CG ^60^.

### Reporting guidelines

Harvard Dataverse: STROBE checklist for ‘The effects of cancer therapies on physical fitness before oesophagogastric cancer surgery: A prospective, blinded, multi-centre, observational, cohort study’. https://doi.org/10.7910/DVN/NUV1OI ^61^.

Data are available under the terms of the Creative Commons Zero "No rights reserved" data waiver (CC0 1.0 Public domain dedication).

## Notes

These data were presented as an oral presentation as part of the EBPOM UK, London, July 2018

**Fit-4-Surgery Consortium Collaborators:** Andrew Bateman ^1, 2^, Timothy J Underwood ^1, 9^, James Byrne ^1, 9^, James Ryan ^7^, Richard Sturgess ^10^, Miland Shrotri ^11^, Mohammed Javed ^11^, Andrew Masters ^11^, Iftikhar Khan ^11^, Adrian Kendrick ^12^

^1^Academic Unit of Cancer Sciences, Faculty of Medicine, University of Southampton, Southampton, UK. SO16 6YD.

^2^Integrative Physiology and Critical Illness Group, Clinical and Experimental Sciences, Faculty of Medicine, University of Southampton, Southampton, UK. SO16 6YD.

^3^Acute Perioperative and Critical Care Research Group, Southampton NIHR Biomedical Research Centre, University Hospital Southampton NHS Foundation Trust, Southampton, UK. SO16 6YD.

^4 ^Department of Statistical Science, University College London, London, UK. W1T 7PJ.

^5 ^Centre for Anaesthesia, Institute of Sport Exercise and Health, University College London Hospitals NIHR Biomedical Research Centre, London, UK. W1T 7HA.

^6 ^Department of Critical Care and Anaesthesia, Lancashire Teaching Hospitals NHS Foundation Trust, Lancashire, UK. PR7 1PP.

^7 ^Department of Critical Care and Anaesthesia, The James Cook University Hospital, Middlesbrough, UK. TS4 3BW.

^8 ^Department of Respiratory Research, University of Liverpool, University Hospitals Aintree, Liverpool, UK. L9 7AL.

^9 ^Department of Upper Gastro-intestinal Surgery, University Hospital Southampton NHS Foundation Trust, Southampton, UK. SO16 6YD.

^10 ^Department of Gastroenterology, University Hospital Aintree, Liverpool, UK. L9 7AL.

^11 ^Department of Surgery, University Hospital Aintree, Liverpool, UK. L9 7AL.

^12 ^Department of Physiological Sciences, University of Bristol, Bristol, UK. BS8 1TH.
