# Supplementary material for: The effects of cancer therapies on physical fitness before oesophagogastric cancer surgery: a prospective, blinded, multi-centre, observational, cohort study
Source: NIHR Open Res. 2021 Jun 16;1:1. [Version 2] doi: 10.3310/nihropenres.13217.1 (PMC7612293; doi:10.3310/nihropenres.13217.1)
Supplement: Peer review history

# Reviewer report: Reviewer response for version 1

**DOI:** 10.3310/nihropenres.14333.r28397

**Published:** 2021-08-18

**Authors:** Juliette Hussey

**Affiliations:** 1 Discipline of Physiotherapy, School of Medicine, Trinity College Dublin, Dublin, Ireland

This is an excellent study which is very clearly presented. I thought the question was clearly defined. The methods are clear and the data analysis appropriate. I could not find any major limitations.

 The paper will add to the scientific research in the area. This is an area of growth and understanding how to improve cardiorespiratory fitness in this cohort is gaining interest.

 The authors are to be commended.

Is the work clearly and accurately presented and does it cite the current literature?

Yes

If applicable, is the statistical analysis and its interpretation appropriate?

Yes

Are all the source data underlying the results available to ensure full reproducibility?

Yes

Is the study design appropriate and is the work technically sound?

Yes

Are the conclusions drawn adequately supported by the results?

Yes

Are sufficient details of methods and analysis provided to allow replication by others?

Yes

Reviewer Expertise:

Exercise in cancer survivorship

I confirm that I have read this submission and believe that I have an appropriate level of expertise to confirm that it is of an acceptable scientific standard.

# Reviewer report: Reviewer response for version 1

**DOI:** 10.3310/nihropenres.14333.r28396

**Published:** 2021-07-02

**Authors:** Javed Sultan, Patrick Casey

**Affiliations:** 1 Department of Oeosphagogastric Surgery, Salford Royal Foundation Trust, Salford, UK; 2 University of Manchester, Manchester, UK

Thank you for the opportunity to review this manuscript produced by leaders in this field.

 The group aimed to validate the hypothesis that neoadjuvant chemoradiotherapy impacted on the physical fitness (defined as VO _2_peak and at AT) of patients with OG cancer. Furthermore, the impact this had on post-operative outcomes and overall survival was also examined. The group clearly demonstrate that most patients become less fit although only one third of patient move risk strata. To this end, the group have fulfilled their primary aim. However, this decline in fitness was not associated with worse survival at 1 and 5 years which, in this relatively small underpowered and self selecting group, is not surprising.

 We congratulate the authors on being able to blind the treating team to the CPET result: a clinically significant piece of information which any surgeon or anaesthetist would be keen to have. I assume this was subject to ethical consideration and cleared.

 The unstandardised chemoradiotherapy regimes is pragmatically understandable but may undermine the results; which the group rightfully highlight as a limitation. We assume the group will also look at the impact of FLOT chemotherapy which is now gold-standard but notably not reported in this study at all.

 Whilst 1 and 5 year survival did not appear different between the comparison groups the fact that only 46% of patients in the high risk group were discharged to their own home and had a much higher re-admission rate is a significant, patient-centred, outcome which in our opinion should be emphasised.

**A single grammatical point noted:**

 In the eligibility criteria paragraph - is the line " *Eligible patients were staged according to a pre-determined protocol" *essentially repeated?

Is the work clearly and accurately presented and does it cite the current literature?

Yes

If applicable, is the statistical analysis and its interpretation appropriate?

Yes

Are all the source data underlying the results available to ensure full reproducibility?

Yes

Is the study design appropriate and is the work technically sound?

Yes

Are the conclusions drawn adequately supported by the results?

Yes

Are sufficient details of methods and analysis provided to allow replication by others?

Yes

Reviewer Expertise:

Oesophagogastric cancer outcomes with regard to preoperative fitness, rehabilitation and muscle mass.

We confirm that we have read this submission and believe that we have an appropriate level of expertise to confirm that it is of an acceptable scientific standard.
